# Retention of Mitochondria in Mature Human Red Blood Cells as the Result of Autophagy Impairment in Rett Syndrome

**DOI:** 10.1038/s41598-017-12069-0

**Published:** 2017-09-26

**Authors:** Diego Sbardella, Grazia Raffaella Tundo, Luisa Campagnolo, Giuseppe Valacchi, Augusto Orlandi, Paolo Curatolo, Giovanna Borsellino, Maurizio D’Esposito, Chiara Ciaccio, Silvia Di Cesare, Donato Di Pierro, Cinzia Galasso, Marta Elena Santarone, Joussef Hayek, Massimiliano Coletta, Stefano Marini

**Affiliations:** 1 Department of Clinical Sciences and Translational Medicine, University of Rome Tor Vergata, Rome, Italy; 2 Department of Biomedicine and Prevention, University of Rome Tor Vergata, Rome, Italy; 3 Department of Life Sciences and Biotechnology, University of Ferrara, Ferrara, Italy; 4 Plant for Human Health Institute, North Carolina State University, Kannapolis, NC USA; 5 Department of Medicine of Systems, University of Tor Vergata, Rome, Italy; 6 Neuroimmunology Unit, Santa Lucia Foundation, Rome, Italy; 7 Institute of Genetics and Biophysics “A.Buzzati Traverso”, Naples, Italy; 8 IRCCS Neuromed, Pozzuoli, (Is) Italy; 9 University Department of Pediatrics, Bambino Gesù Children’s Hospital, University of Rome Tor Vergata, Rome, Italy; 10 Child Neuropsychiatry Unit, University Hospital, Azienda Ospedaliera Universitaria Senese (AOUS), Siena, Italy

## Abstract

Rett Syndrome (RTT), which affects approximately 1:10.000 live births, is a X-linked pervasive neuro-developmental disorder which is caused, in the vast majority of cases, by a sporadic mutation in the Methyl-CpG-binding protein-2 (MeCP2) gene. This is a transcriptional activator/repressor with presumed pleiotropic activities. The broad tissue expression of MeCP2 suggests that it may be involved in several metabolic pathways, but the molecular mechanisms which provoke the onset and progression of the syndrome are largely unknown. In this paper, we report that primary fibroblasts that have been isolated from RTT patients display a defective formation of autophagosomes under conditions of nutrient starvation and that the mature Red Blood Cells of some RTT patients retain mitochondria. Moreover, we provide evidence regarding the accumulation of the p62/SQSTM1 protein and ubiquitin-aggregated structures in the cerebellum of *Mecp2* knockout mouse model (*Mecp2* ^−/*y*^) during transition from the non-symptomatic to the symptomatic stage of the disease. Hence, we propose that a defective autophagy could be involved in the RTT clinical phenotype, which introduces new molecular perspectives in the pathogenesis of the syndrome.

## Introduction

Rett Syndrome (RTT) is a X-linked pervasive neuro-developmental disorder affecting approximately 1:10.000 live births which was originally classified as an autism-related disorder with abnormalities linked to the Central Nervous System (CNS). Within the first 6–12 months of life, individuals with RTT develop progressive intellectual and motor disabilities that strongly limit life expectancy^1,2^. Due to the broad expression of the Methyl-CpG-binding protein-2 (MeCP2) gene, which is mutated in the 90–95% of patients (while the remaining 5–10% carry mutations either in the CDKL-5 or in FOXG1–2 genes), there have been several clinical and biochemical observations demonstrating that MeCP-2 mutations can lead to abnormalities and dysfunction in several tissues^3–22^. MeCP2 is a transcriptional activator/repressor which is thought to be involved in pleiotropic activities, but exactly how the spectrum of MeCP-2 mutations leads to RTT phenotypes is unclear and unfortunately, to date, no cure for the disease exists^1,2^.

A preliminary set of data in our lab showed: (*i*) a reduced ability to grow in a low-nutrient medium of primary skin fibroblasts (hereafter referred to as fibroblasts) that had been isolated from RTT patients (unpublished observation); (*ii*) an increase in intracellular Reactive Oxygen Species (ROS) and a reduced ATP content in fibroblasts and also in RBCs harvested from RTT patients^23^. On the basis of these data, we investigated the major intracellular proteolytic pathway which regulates homeostasis under low-nutrient and other stressful (*i*.*e*. heat and oxidative stress, infections) conditions, *i*.*e*. autophagy and, in particular, macroautophagy^24–30^.

Macroautophagy is a tightly regulated process which is triggered under stressful conditions, such as nutrient starvation, whereby cells engulf in a double membrane vesicle (*i*.*e*., autophagosomes) unnecessary/damaged organelles or cytosolic components that should be delivered to lysosomes for degradation and the recycling of nutrients^24–30^.

It is interesting to note that autophagy also plays a key role in physiological processes, such as axon sprouting and dendritic spine arborisation during CNS development as well as clearance of mitochondria during reticulocyte maturation to RBCs^31–34^.

In this paper, we describe a defective autophagy in RTT patients. This discovery is supported by evidence obtained in primary skin fibroblasts and RBCs isolated from human patientsand by monitoring the accumulation of autophagy-markers in the cerebellum of a murine model of the disease.

## Results and Discussion

### RTT fibroblasts show a defective autophagy activation under starving conditions

In order to verify the hypothesis regarding a defective autophagy in RTT patients, we analyzed autophagy activation under starving conditions in skin primary fibroblasts isolated from RTT patients (n = 4) and healthy subjects (n = 4)^17,19^.

**Figure 1:**
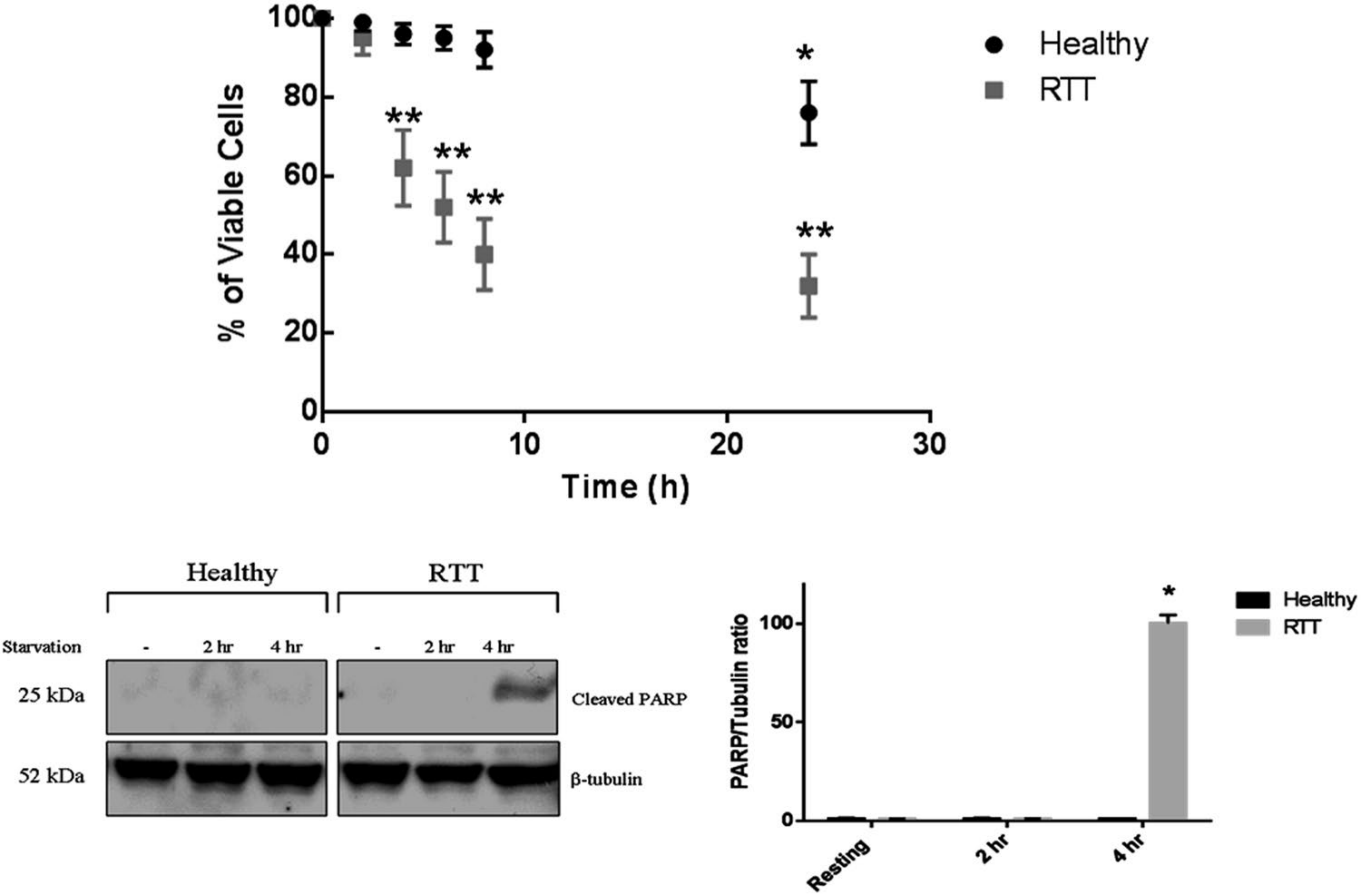
Decreased viability of RTT fibroblasts in starvation medium. (**a**) Time-dependent viability of RTT and healthy fibroblasts cultivated over 24 h in the starvation medium. Results are reported as percentage of living cells *vs* the number of cells at time 0 of the assay. Results presented are the means ± S.E. of five independent experiments performed in triplicate. *Significantly different from healthy cells at time 0; **significantly different from either RTT cells time 0 and from the viability of healthy cells at the corresponding time point (*p < 0.01, **p < 0.05, oneway ANOVA, followed by Tukey’s test, n = 15). (**b**) Western blotting analysis of the p25 fragment of PARP (Poly (ADP-ribose) polymerase (PARP), a family of proteins involved in a number of cellular processes concerning mainly DNA repair and programmed cell death) in RTT and healthy fibroblasts grown under starving condition for 2 and 4 h. Beta-tubulin was used as internal control. A representative immunoblot of three independent experiments is reported. Results presented are the means ± S.E. of three independent experiments performed in triplicate. *Significantly different from control (either healthy and RTT cells at time 0) (*p < 0.001, one-way ANOVA, followed by Tukey’s test, n = 9).

We first defined the viability over time of healthy and RTT fibroblasts cultured in starvation medium by both MTT and Trypan blue exclusion tests. With respect to the viability of RTT fibroblasts grown in standard medium, RTT fibroblast viability decreased after 4 h of starvation (20 ± 3.3% of dying cells, p < 0.05); this percentage considerably increased after 6–8 h of starvation (60 ± 5.1% of dying cells, p < 0.05) (Fig. 1a). Conversely, healthy fibroblasts were still fully viable after 4 h of starvation with only a very low reduction in viability after 6–8 h (10 ± 1.2% of dying cells, p < 0.01) with respect to the viability of fibroblasts grown in standard medium (Fig. 1a). Thus, as from a direct comparison of the viability of the two cell lines at each time-point, it came out that the viability of RTT fibroblasts was severely compromised starting from time 4 h (see details in the figure legends, p < 0.05). Moreover, a Western blotting (WB) analysis highlighted that in RTT fibroblasts which had been starved for 4 h, a band corresponding to the p25 fragment of the poly (ADP-ribose)polymerase-1 (PARP) was greatly increased (96 ± 4%, p < 0.001) with respect to the faint detection at time 0. This fragment is released by the caspase-dependent degradation of the protein during the early phase of apoptosis induction (Fig. 1b)^35^. The p25 fragment was almost undetectable in the healthy fibroblasts under the same experimental conditions.

These data confirmed that RTT fibroblasts did not tolerate growth in a starving medium, and quickly underwent apoptosis. Therefore, we further investigated the autophagic flux by monitoring the LC3B-II marker clearance in the presence and in the absence of a lysosome-inhibitor, such as chloroquine (CQ)^24–30^.

**Figure 2:**
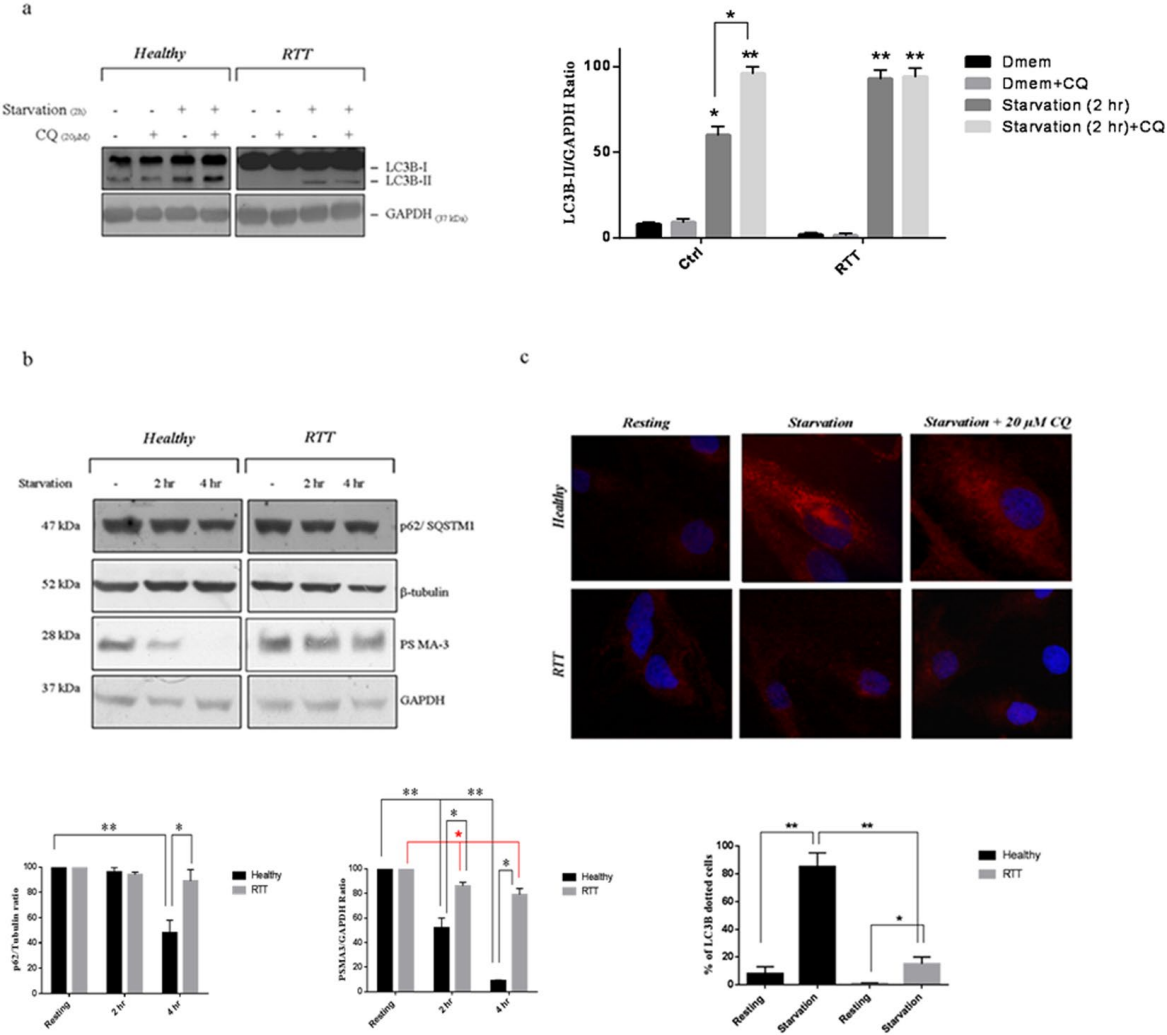
Defective autophagy activation in RTT fibroblasts. (**a**) WB analysis of RTT and healthy fibroblasts cultivated either in DMEM or starvation medium (2 h) in the presence or absence of 20 µM of CQ (*upper panel*). Filters were probed with an anti-LC3B or an anti-GAPDH antibody. A representative immunoblot of three independent experiments performed on the cell lines available (n = 4 for both the healthy and the RTT fibroblasts) is reported. Densitometric determination of LC3B to GAPDH content was performed by ImageJ software (*lower panel*). Results are the means ± S.E. of three independent experiments. Differences between the different experimental conditions in the same group are significantly different (*p < 0.05; **p < 0.001, one-way ANOVA, followed by Tukey’s test, *n* = 32). (**b**) Healthy and RTT fibroblasts (n = 4 in both cases) cultivated in a starvation medium for 2 and 4 h in the absence of CQ were assayed for p62/SQSTM1 and PSMA-3 content by WB (*upper panel*). The average intensity of the band was normalized to that of β-tubulin (for p62) and GAPDH (for PSMA-3) by ImageJ software (*lower panel*). The image shown is representative of four independent experiments. Even though the viability of RTT fibroblasts at 4 h of starvation was already compromised (see Fig. 1), this time-point was necessary to follow degradation of p62 which is normally delayed^32^. The results are the means ± S.E. of four independent experiments performed in triplicate. ^*,**,*^Significantly different from the specific (see bars) experimental condition (^*,*^ *p* < 0.05; **p < 0.001 one-way ANOVA; followed by Tukey’s test, *n* = 24). (**c**) Immunofluorescence microscopy analysis of autophagy activation by healthy (*upper panel*) and RTT fibroblasts (*lower panel*) (n = 4 in both cases). Resting (*left*), starved (*middle*) and starved + 20 µM of CQ (*right*) cells were stained with an anti-LC3B antibody. Due to the low detection of autophagosomes under resting conditions, the cells positive for autophagy induction under starving condition were quantified as the percentage of those displaying at least 10 dots. The results are the means ± S.E. of three independent experiments. **Significantly different from the specific (see bars) experimental condition (*p < 0.001; **p < 0.005, Unpaired τ Student’s test, n = 24).

After having ruled out a CQ-linked cytotoxic effect (Supplementary Fig. S1), healthy and RTT fibroblasts were cultivated either in resting or in starvation media for 2 h in the presence or absence of 20 µM of CQ to quantify the LC3B-II/GAPDH ratio by WB (Fig. 2a). The pattern of the LC3B-II/GAPDH ratio (or any other internal control which is not modulated by autophagy) under conditions of autophagy activation and in the presence and in the absence of CQ (or any other lysosomal inhibitor) is considered a valid strategy to monitor the autophagy flux.

In healthy fibroblasts, cultivated in standard medium, LC3B-I was clearly immuno-detected, whereas LC3B-II was faint. The LC3BII/GAPDH ratio of healthy fibroblasts cultivated in standard medium in the absence and in the presence of CQ was comparable (Fig. 2a, *left panel*). This observation suggests that the basal autophagy was almost undetectable in the healthy fibroblasts (Fig. 2a, *left panel*).

When these cells were grown in the starvation medium and in the absence of CQ for 2 h, the LC3B-II/GAPDH ratio increased (60 ± 4.8%, p < 0.05) compared to that of healthy fibroblasts cultivated in standard medium either in the absence or the presence of CQ (which was, as previously mentioned, identical) (Fig. 2a, *left and right panel*). This behaviour, indeed, indicates that LC3-I actually underwent lipidation to form LC3B-II, which is an early marker of autophagy activation^24–30^. In order to test the presence of this autophagy activation, we cultivated cells in a starvation medium in the presence of CQ for 2 h and they displayed a further increase of the LC3B-II/GAPDH ratio (94 ± 5.5%, p < 0.001) with respect of healthy fibroblasts cultivated in standard medium (Fig. 2a, *left and right panel*).

Furthermore, the difference between the LC3B-II/GAPDH ratio of healthy fibroblasts grown in starvation medium in the absence and in the presence of CQ was statistically significant (p < 0.05, Fig. 2a, *left panel*)

Thus, according to the standard interpretation of the LC3B-II pattern by WB analysis, the overall result indicates that, in the absence of nutrients, healthy fibroblasts stimulate the formation of autophagosomes which undergo accumulation in the presence of CQ. This behaviour is compatible with a normal autophagic flux^24–30^. Interestingly, in the case of RTT fibroblasts grown in standard medium, while LC3B-I was clearly immuno-detected, LC3B-II was very faint in cells both in the absence and in the presence of CQ (Fig. 2a, *left panel*). Under starving conditions and in the absence of CQ, the appearance of a faint band corresponding to LC3B-II in RTT fibroblasts only after long exposure of the filter suggested that at least a minimal lipidation of LC3B-I occurred (Fig. 2a, *left panel*). Hence, the LC3B-II/GAPDH ratio between RTT fibroblasts grown in starvation medium in the absence of CQ was increased (93 ± 5.5%, p < 0.001) with respect to that of RTT fibroblasts grown in standard medium in the absence of CQ (Fig. 2a, *right panel*).

However, the administration of CQ to RTT fibroblasts grown in starvation medium did not further increase the LC3B-II/GAPDH ratio which was increased (95 ± 3.5%, p < 0.001) if compared to cells grown in standard medium in the absence of CQ, but was not significantly increased with respect to the LC3B-II/GAPDH ratio of RTT fibroblasts grown in starvation medium in the absence of CQ (Fig. 2a, *left panel*). This finding suggested that no accumulation of autophagosomes occurs in RTT cells and the WB analysis supported a block, at some level, in the autophagy flux^24–31^.

To further strengthen our hypothesis concerning a possible defective autophagic flux, we verified the degradation of two recognized autophagy reporter substrates in healthy and RTT fibroblasts starved for 2 and 4 h in the absence of CQ, namely: (*i*) the p62/SQSMT1 protein, which assists the clearance of poly-ubiquitinated protein aggregates and is itself degraded by the lysosomal hydrolases^36^; (*ii*) the 20 S proteasome, which is quickly degraded under starving conditions^37,38^. With respect to the basal level of the two proteins (*i*.*e*. time 0), a decrease over time of the p62/tubulin ratio (44 ± 10% after 4 h, p < 0.001) and of the PSMA-3/GAPDH ratio (*i*.*e*. the α7 subunit of the 20 S proteasome) (45 ± 3.1% after 2 h, 11 ± 5.2% after 4 h, in both cases p < 0.001) was observed in healthy fibroblasts (Fig. 2b, *upper panel*).

In the case of RTT fibroblasts, the decrease of the p62/tubulin ratio over time was not statistically significant even after 4 h (89 ± 9%), whereas the decrease of the PSMA3/GAPDH ratio at 2 h (81 ± 4.4%, p < 0.05) and 4 h (78 ± 5.1%, p < 0.05) was significantly different if compared with the basal level of the protein (*i*.*e*. time 0) (Fig. 2b, *lower panel*). However, differences between the healthy and RTT groups were statistically significant in the case of the p62/tubulin ratio only at 4 h (p < 0.05), whereas the PSMA3/GAPDH ratio was significantly different between the experimental groups both at 2 h (p < 0.05) and 4 h (p < 0.001) (Fig. 2b, *lower panel*).

Interestingly, the β-tubulin and GAPDH patterns, used as internal controls for the p62 staining and the PSMA3 staining, respectively, were unaltered over 4 h of starvation in healthy fibroblasts. However, the intensity of the β-tubulin band, as well as that of the GAPDH band, decreased significantly in RTT fibroblasts starved for 4 h compared to what was observed at time 0 or time 2 h for these cells (Fig. 2b, *higher panel*). This reduction was probably due to the lower number of living RTT fibroblasts harvested at this time point (in accord with the poor viability of RTT fibroblasts at 4 h of starvation reported in Fig. 1a) and not due to a down-regulation of β-tubulin over time. As a whole, it was found that RTT fibroblasts did not efficiently degrade these autophagy reporter substrates, supporting the hypothesis of a defective autophagy.

To cast further light on the features of this block, we performed an immuno-fluorescence analysis by using the LC3B antibody. We analysed healthy and RTT fibroblasts grown in standard medium in the absence of CQ (resting condition) and in the starvation medium for 2 h in the presence or absence of 20 µM CQ (Fig. 2c). Consistent with the slow metabolic rate of the primary cells, we found that resting healthy and RTT fibroblasts displayed a weak and diffuse LC3B^+^ staining with a low percentage of cells showing more that 10 dots (*i*.*e*. autophagosomes) (8 ± 5% and 1 ± 0.5%, respectively) indicating that, by immuno-fluorescence, the basal autophagy was poorly detectable in accord with the data reported in Fig. 2a.

With respect to cells grown in standard medium, the percentage of healthy fibroblasts grown in starvation medium (in the absence of CQ) displaying at least 10 LC3B^+^ autophagosomes was markedly increased (82 ± 10%, p < 0.005), whereas RTT fibroblasts grown in starvation medium showed a slight increase (17 ± 8%, p < 0.001). As a matter of fact the percentage of healthy fibroblasts grown in starvation medium displaying at least 10 autophagosome was significantly higher than that of RTT fibroblasts grown under the same experimental condition (p < 0.005). The autophagosomes were mainly localized to the perinuclear region. In the presence of CQ an expected diffuse and intense LC3B^+^ staining was further observed, even though in this case, the diffuse staining did not allow to precisely quantify the number of dots.

Administration of CQ was ineffective in RTT fibroblasts, clearly indicating a severely impaired autophagosome biogenesis (Fig. 2c).

Given the relevance of the putative impairment on autophagosome biogenesis, an additional approach was adopted to confirm this observation. Both healthy and RTT fibroblasts, starved for 2 h in the absence of CQ, were stained with a specific dye (Cyto-ID) for the autophagosomal membrane and analyzed by immuno-fluorescence. Even in this case, while in healthy fibroblasts several autophagosomes were actually detected, in RTT fibroblasts only a limited number of small and isolated vesicles was observed (Supplementary Fig. S2). The difference in the percentage of cells displaying at least 5 Cyto-ID positive dots between healthy and RTT fibroblasts was markedly significant (80 ± 4% *vs* 8 ± 6%, respectively, p < 0.001). Therefore, the overall analysis provided evidence that a defective autophagosome biogenesis occurs in RTT primary fibroblasts.

### Mature RBCs of RTT patients carrying the R255X MeCP2 mutation retain mitochondria

We next sought to determine whether additional signs of defective autophagy could be observed *ex vivo* in RTT patients. Due to the fact that mitochondria clearance is a classic autophagy-based mechanism occurring in circulating reticulocytes at the final stage of maturation to RBCs^32–34^ we extended our investigation also to *ex vivo* human RBCs to verify whether mitochondria are retained in these cells.

**Figure 3:**
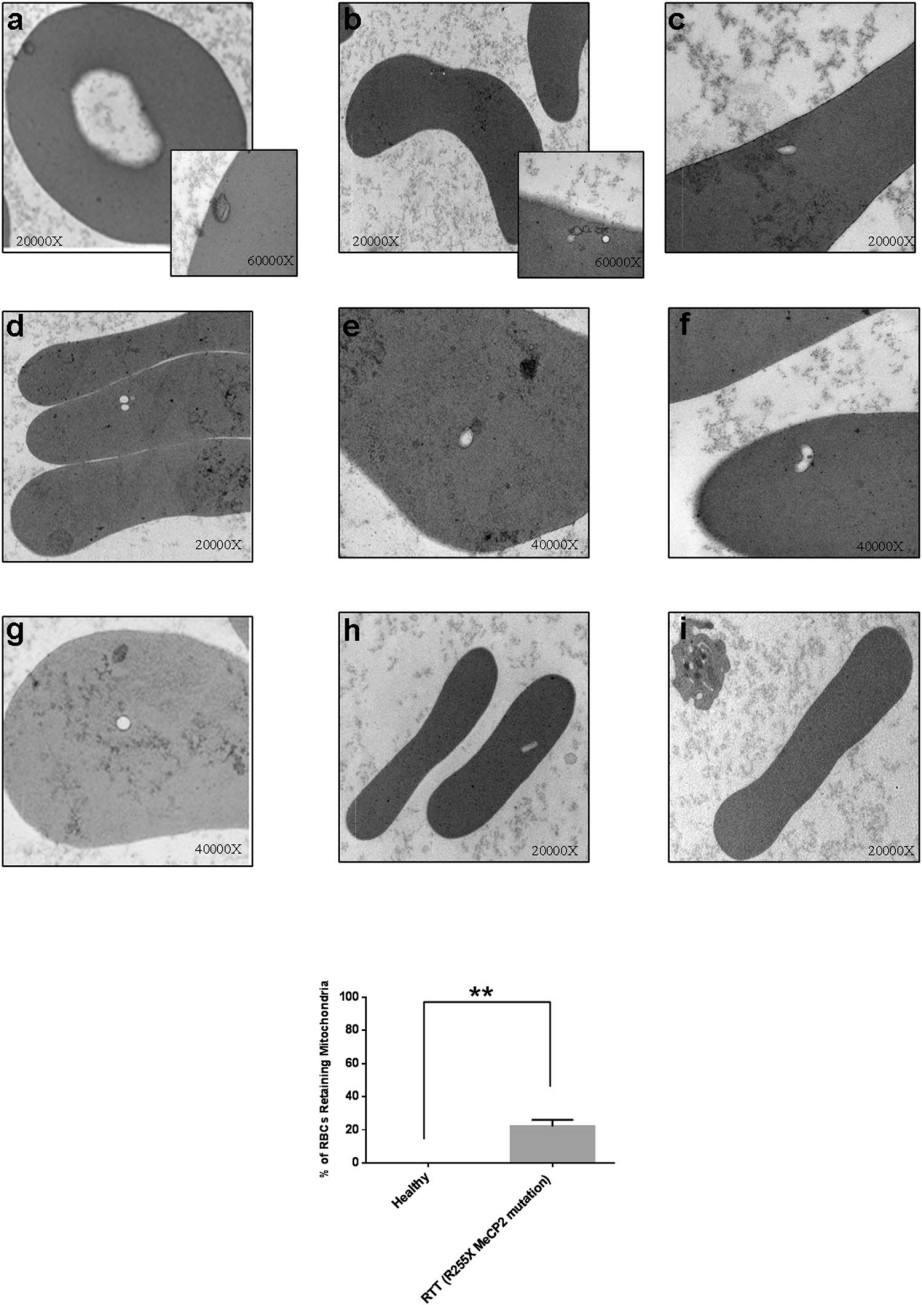
TEM acquisition showing mitochondria in mature RBCs of RTT patients. *Upper panel*: Transmission Electron Microscopy acquisitions of RTT (n = 3) and healthy (n = 3) (*bottom right*) patients’ RBCs. Structures Resembling Mitochondria (SRM) of different shapes were observed with a significant frequency in RTT RBCs of patients bearing the R255X MCP2 mutations (a total of 3 out of 15 subjects) (**a**–**h**). These structures were not detected in healthy patients (i). Images were acquired at different magnification ranging from 20,000× to 60,000×. *Lower panel*: Statistical analysis. The number of RBCs displaying at least one SRM were counted in 10 different fields. The differences were statistically significant for p < 0.0004 (Unpaired τ Student’s test).

Hence, RBCs from RTT patients (n = 15) and from healthy donors (n = 11) were analyzed by transmission electron microscopy (TEM). The TEM investigation highlighted the presence of structures resembling mitochondria (SRM) in bi-concave shaped RBCs isolated from the majority of the RTT patients (11 out of 15). Among those 11 RTT patients, three of them displayed the most severe cohort of symptoms and also a high frequency of RBCs (20 ± 4%) (Fig. 3a–h). As expected, SRM were undetectable in healthy RBCs (Fig. 3I). Differences between healthy and RTT subjects were statistically significant (p < 0.0004; Fig. 3, *lower panel*).

Nonetheless, the severity of the symptoms was based on the clinical assessment and, accordingly, those three patients with the most severe of symptoms all bore the R255X mutation of the MeCP2 gene which is associated to a ﻿severe prognosis^39^.

**Figure 4:**
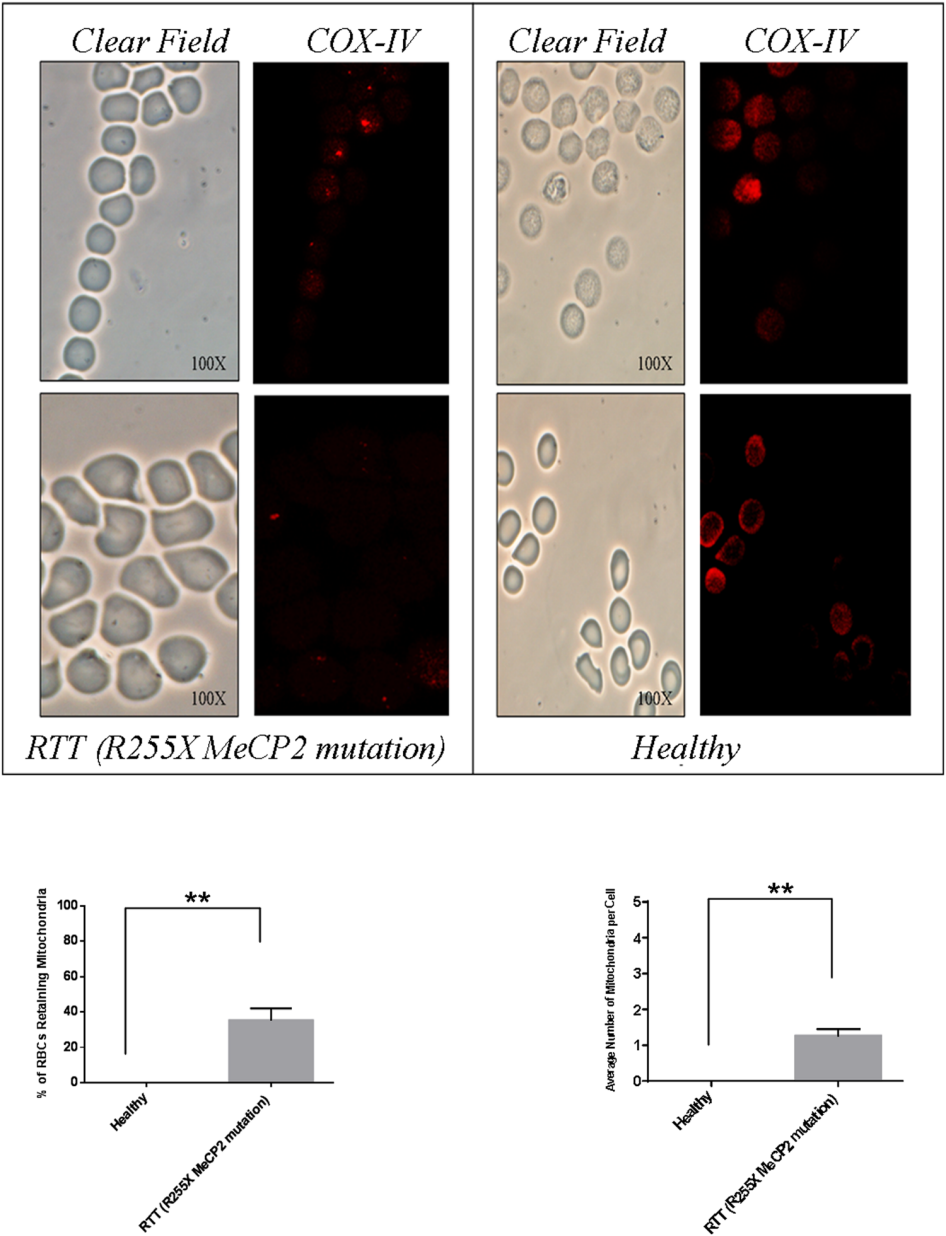
Identification of mitochondria in mature RBCs of RTT patients by IF. *Upper panel*: Immunofluorescence microscopy analysis of the RBCs isolated from the RTT patients carrying the R255X MeCP2 mutation (n = 3) (*left panel*) and from healthy individuals (n = 3) (*right panel*). RBCs were allowed to adhere to glasses by cytospinning and were probed with an anti COX-IV antibody. RTT RBCs displayed a COX IV^+^ dotted pattern that was undetectable in healthy RBCs. Clear field acquisition highlighted a normal bi-concave shape of the RBCs. The experiment was carried out in triplicate by using the same blood specimen. *Lower panel*: the percentage of RBCs displaying at least 1 mitochondria was calculated in 10 different fields (*left panel*); the average number of mitochondria per RBC was calculated by counting the number of dots for each RBC in the different fields (*right panel*). The results are the means ± S.E.; **significantly different from control (**p < 0.005, unpaired τ Student’s test).

As a consequence and in consideration of the fact that we had no possibility to enroll a higher number of subjects harbouring other mutations whit a low or null frequency of mitochondria-retaining RBCs, we focused our attention on the analysis of those three patients. TEM analysis documented, in those three above-mentioned cases, the presence of structures resembling intact or partially digested mitochondria (either electron-dense or lucent), which were normally or dumbbell (elongated) shaped, and generally small with faint cristae (Fig. 3a–h). In fact, the size and the overall shape of these structures were found to be be consistent with those of mitochondria retained into mature RBCs in non-RTT murine models of defective macroautophagy (*i*.*e*., the Ulk1^−/−^ mice) or mitophagy (*i*.*e*. the Nix^−/−^ mice) reported by other authors^32,33^. Interestingly, the morphological alterations of mitochondria we observed, such as the reduced dimension, the elongated (*i*.*e*. dumbbell shaped) structure and, in particular, the presence of faint cristae have already been described in the muscle and in the cerebellum of RTT patients^20,21^. To confirm the mitochondrial identity of the SRM, we stained RBCs from healthy donors and these RTT patients with severe symptoms with an anti-COX-IV (cytochrome c oxidase) antibody. A statistically significant number of RBCs from the three RTT patients in comparison to RBCs of healthy subjects(35 ± 5% *vs* 0.2 ± 0.01%, respectively, p < 0.005) displayed a COX-IV^+^ dotted pattern which suggested mitochondria retention (Fig. 4). The average content of mitochondria per cell was calculated to be 1.2 ± 0.2 organelles per RBC in RTT patients and 0.002 ± 0.0002 in healthy subjects (p < 0.005) (Fig. 4). Differences in percentage of RBCs displaying mitochondria between TEM and the IF investigations can be put down to the fact that the possibility of detecting the organelles by TEM is strictly dependent on the thin section of the cell, which can hinder their presence, whereas the IF approach is not limited in this way.

To further confirm those results, we performed a cytofluorimetric analysis of the RBCs of both healthy subjects and the three RTT patients by using an anti-CD71 (transferrin receptor) and anti-COX-IV antibodies. The frequency of CD71 positivity, a marker of immature RBCs, was not significantly different in healthy and RTT patients (1.34 ± 0.13% *vs* 1.03 ± 0.3%; Supplementary Fig. S3). It should be underlined that the RTT patients included in the study displayed normal haematological parameters and, even though the reticulocyte index was, at least in a couple of subjects, lower than that found in healthy subjects, the overall differences between the two patients groups were not statistically significant (Table 1). Conversely, a very faint COX-IV^+^ population was observed in healthy RBCs, whereas a higher frequency of COX-IV^+^ cells was detected in RTT RBCs (0.056 ± 0.004% *vs* 1.15 ± 0.19%, respectively, p < 0.01). These results were further confirmed by using Mitotracker Green (MT) staining (Supplementary Fig. S3), that documented an increase of MT^+^ RBCs in one RTT patient (bearing the R255X MeCP2 mutation) *vs* one healthy patients (0.37% *vs* 0.16%), similar to that observed with the COX-IV antibody previously described.

**Table 1:** Haematological parameters of the patients involved in the study.

	Healthy	RTT
RBC (10^6^/µL)	5.45 ± 0.5	5 ± 0.6
HGB (g/dL)	16.5 ± 0.8	15.5 ± 1.5
HCT (%)	49.8 ± 1	46.2 ± 3
MCV (fL)	92 ± 4	85 ± 6
RI (%)	1.81 ± 0.3	1.77 ± 0.5

Results are presented as the mean ± SEM of the patients analyzed in the study. The parameters were obtained through collaboration with the Clinical Biochemistry Unit of the Tor Vergata Hospital.

Notably, the differences were found to be not-statistically significant by unpaired Student’s test.

In order to further validate the identity of SRM, an equal number of RBCs was lysed and analyzed by WB under denaturing and reducing conditions. Filters were stained with antibodies against sirtuin-3, a de-acetylase specifically expressed in the mitochondrial matrix^40^. Sirtuin3 was chosen as a mitochondrial marker in this approach because, differently from COX-IV and other mitochondrial markers, its molecular weight doesn’t overlap with that of haemoglobin chains or haemoglobin oligomers in the electrophoretic pattern. In the three RTT patients examined, a statistically significant increase of the sirtuin-3/GAPDH ratio was observed for each patient with respect to the same ratio in lysates from healthy RBCs (p < 0. 001) (Supplementary Fig. S4).

Those three patients, all bearing the MeCP2 mutation (*i*.*e*., R255X), displayed the worst phenotype, with a very high percentage of RBCs displaying at least 1 mitochondria. However, the very limited number of patients at our disposal made it difficult to draw any statistically significant conclusion regarding the possibility of a relationship between the severity of the disease and the extent of mitochondrial retention inside mature RBCs. Another point, which appears to be worth mentioning, is that retention of mitochondria inside mature RBCs in the previously cited Ulk1^−/−^ and Nix^−/−^ murine models led to anaemia or other blood pathologies likely determined by the increased clearance of these abnormal erythrocytes by spleen macrophages^32,33^. In the RTT patients enrolled in this study, only a very limited and not statistically significant decrease in haematological values was registered, and they seemed not to be anaemic or to suffer of other blood pathologies. The discrepancy between our results and those from the murine models could be due to the fact that the knock-down of macroautophagy or mitophagy genes (*i*.*e*. Ulk1^−/−^ and Nix^−/−^ mice, respectively) induces a very high percentage of mitochondria-retaining RBCs and the retention of several organelles within RBCs, which is considerably a more severe defect compared with that documented in RTT patients^32,33^. Even though we observed a significantly high number of mitochondria-retaining RBCs in patients carrying the R255X mutation (Fig. 4), the number of organelles in each cell was very low in RTT RBCs (Fig. 4). This might not be enough to drive major morphological and functional alterations in RBCs. It is worth mentioning that, although the mitochondrial retention inside RBCs could be at least one factor determining the severe redox imbalance observed in RTT RBCs in association with a significant change of energy state and metabolism (*i*.*e*., ATP/ADP and NADH/NAD ratio), recent studies reported that the kinetics of oxygen bindings to haemoglobin and oxygen diffusion almost overlap those of healthy patients^16,17,23,41^.

The apparent discrepancy between our results and those deriving from murine models might also find an explanation in the fact that the RTT syndrome is an X-linked disorder that affects almost exclusively the female gender and inactivation of one of the two copies of the X-chromosome (XCI) is a random phenomenon occurring during embryogenesis, as widely documented^42,43^. Thus, in the case of the RTT syndrome, the random XCI would be expected to produce, in somatic cells, a mosaicism with half of the cells expressing the *wild-type allele*, with the remaining half expressing the mutated allele of the MeCP2 gene. Interestingly, the possibility that at least some MeCP2 mutations could be associated with a non-random XCI in the neuronal tissue is in agreement with the phenotypical variability of RTT patients (and also for the familiar cases of RTT), a characteristic which has been extensively documented^42,43^. Theoretically, if half of the haematological progenitors in the bone marrow retain the X chromosome bearing the MeCP2 mutation, only half of the circulating mature RBCs would carry the pathological allele, thus restricting the number of circulating mitochondria-retaining RBCs. Nevertheless, an increased prevalence of non-random XCI in favour of the *wild-type* allele has been observed in circulating leukocytes in sporadic RTT patients^42,43^. Regarding this point, it would be very challenging to address whether the patients that display or not a low number of mitochondria-retaining RBCs also display a less significant impairment of mitophagy or the non-random XCI, which would lead to the positive selection of haematological progenitors bearing the *wild-type* MeCP2 allele.

Hence, given the complexity of the RTT pathology, we prefer to assert that the RTT patients, together with the patients affected by the Pearson Syndrome, could represent the first cases of mitochondria retention in human mature RBCs^44^.

### Evidence of defective autophagy in the cerebellum of Mecp2-null mice

**Figure 5:**
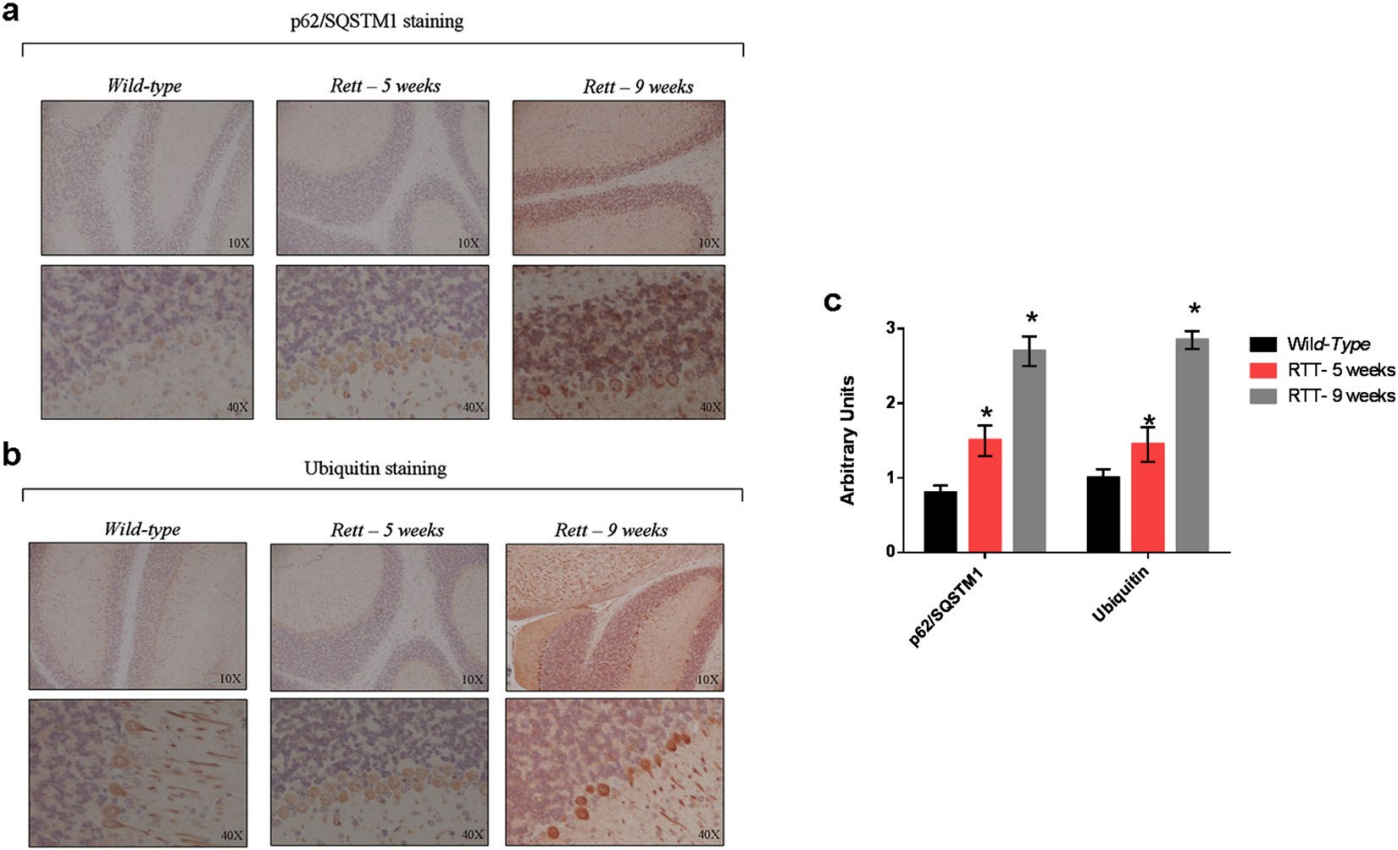
Accumulation of autophagy reporter substrates in the cerebellum of murine models of RTT. Immunohistochemistry analysis of the cerebellum from 9-weeks-old *wild-type* mice and 5- (asymptomatic) and 9-weeks (symptomatic) old RTT mice knock-out for MeCP2 (n = 3 for each experimental group). For the three experimental conditions, the organs isolated from the different animals were studied. Slices (n = 4) from each organ were probed with anti-p62/SQSTM1 (**a**) and an anti-Ub antibody (**b**). An age-linear increase in the staining for both p62/SQSTM1 and Ub was observed in all the cerebellum layers of RTT mice compared to the cerebellum of *wild-type* animals. (**c**) Bar graph showing the semiquantitative evaluation of p62/SQSTM1 and Ub immunoreactivity, expressed as arbitrary unit. The results were expressed as mean number ± S.E., with an interobserver reproducibility of ±95%. The staining of the cerebellum of 5-weeks mice was compared to that of *wild-type* mice, and the staining of the 9-weeks mice to that of 5-weeks mice. Differences were evaluated by a Student’s *τ* test and were considered significant at p value ≤ 0.05.

Given that RTT is a neurodevelopmental disorder, in order to further support our results which point toward a systemic defective autophagy, we performed immuno-histochemical analyses for p62 and ubiquitin of the cerebellum (*i*.*e*. an organ in which mitochondria alterations and other major histological abnormalities have been observed)^21,45^ of 9-weeks-old *wild-type* mice and 5-weeks-old (asymptomatic) and 9-weeks-old (symptomatic) *Mecp2* ^−/*y*^ mice. For each experimental condition, the organ isolated from three animals was analyzed. Compared to *wt*, RTT cerebellum showed an increase in the intensity of p62 (Fig. 5a) and Ub (Fig. 5b) staining in all layers (*i*.*e*., granules, Purkinje and cortical layers), which was linear with the animals age. Moreover, the hyper-stained structures resembled intracellular aggregates. According to the semiquantitative evaluation of the intensity of the staining of either p62/SQSTM1 and Ub, differences between the cerebellum of 5-weeks animals *vs* healthy animals, and of 9-weeks animals *vs* 5-weeks animals were statistically significant (p < 0.05).

The histological difference between asymptomatic and symptomatic animals suggests that the autophagy alteration could be absent or weak at birth, while it progressively enhances during the first weeks of life (and possibly when the MeCP2 activity reaches a peak), giving rise to the symptoms.

## Conclusions

A putative autophagy impairment has been previously suggested (but not explored) in non-syndromic autism as a consequence of an increased mTOR signalling which physiologically blocks autophagy activation. However, in RTT, there is contradictory evidence which indicates a decrease in mTOR signalling^13,46^.

The biological rationale behind our evidence of systemic autophagy impairment in RTT patients carrying the MeCP2 mutation is difficult to interpret because the MeCP2 nuclear role remains unclear and few genes whose expression is altered in MeCP2 mutated tissues have been reported. Nonetheless, it is, in any case, important to recall that each MeCP2 mutation (and a myriad of different mutations has been reported) is potentially associated with a different set of genes whose transcription is altered. However, recently, Nott and co-workers highlighted that MeCP2 positively regulates the transcriptional activity of FOXO, which is a major factor in autophagy gene expression^47^. Although further studies are required to address the role of FOXO in our systems, this observation is the only molecular detail to date available which is in line with our hypothesis that a systemic defective autophagy could be one of the driving causes of the RTT syndrome phenotype. Therefore, we must emphasize that, at this stage, we are unable to prove that the molecular defect observed is directly caused by the MeCP2 mutation.

In conclusion, in this paper, we describe an unprecedented retention of organelles in the mature RBCs of human subjects and we cast light on autophagy, which is a central metabolic pathway in cell biology, whose dysregulation could characterize the RTT syndrome onset and progression, thus paving the road to strategies of therapeutic intervention.

## Materials and Methods

### Autophagy induction in fibroblasts

RTT fibroblasts were isolated from patients as previously indicated^17,19^. Rett patients bore the following MeCP2 mutations: R168X, P152R, R190 (frame-shift), R255X. Normal fibroblasts were harvested from healthy subjects and then matched for age and gender. Healthy and RTT fibroblasts were grown in DMEM supplemented with 10% FBS. To induce autophagy, an equal number of cells (*i*.*e*. 5 × 10^5^) was seeded the day before the experiment and, in the case of the starved cell, the cell mono-layer was washed once with pre-warmed PBS and twice with starvation medium (140 mM NaCl, 1 mM CaCl_2_, 1 mM MgCl_2_, 5 mM D-Glucose, 20 mM Hepes, 1% BSA, pH 7.4). Thereafter, cells were incubated for the indicated time in the starvation medium in the presence or absence of 20 μM chloroquine (CQ). Whenever the cells were not grown in the starvation medium, the medium was replaced with standard medium supplemented or not with 20 μM CQ. Of note, different starvation media were used (EBSS, HBSS), but the buffer we used provided the strongest induction in healthy fibroblasts in accordance with findings from other authors^48^.

The cells from each experimental condition were then harvested after 2 h of incubation and lysed (25 mM Hepes, 0.1% SDS, 1% NP-40, 1 mM EDTA, 0.1% DTT, pH 7.4 supplemented with protease and phosphatase inhibitor cocktails) on ice. The supernatant was then cleared by centrifugation at 13.000 rpm for 30 min at 4 °C and the proteins were separated through a 15% (or 12% depending on the protein of interest) acryl-amide gel. Proteins were then blotted onto a PVDF or nitrocellulose filter and probed with the following antibodies: anti-LC3B (raised against the N-terminal, thus bearing higher affinity for LC3B-II than for LC3B-I) (Cell Signaling Technologies); anti-p62/SQSTM1, anti-PSMA-3; anti-tubulin; anti-GAPDH (Abcam, CO, UK).

LC3B-I (Microtubule associated protein 1A/3B-light chain) is a cytosolic protein involved in the early steps of autophagosome biogenesis. During autophagy activation, LC3B-I is labelled with a phosphatidylethanolammine (PE) tail, forming LC3B-II. This protein is then recruited by the autophagosomal membrane where it mediates membrane expansion and fusion with lysosomes.

LC3B-II degradation during the autophagic process is therefore a reliable method to assess the formation and the degradation of the autophagosomes^23,24,30–32^.

However, it is important to bear in mind that LC3B-I lipidation also occurs at non-autophagosomal sites: hence, the increase in LC3B-II must be compared to that of cells starved over the same time in the presence of a lysosome inhibitor, such as chloroquine (CQ) (Sigma-Aldrich, CO, St. Louis)^30,31^.

Densitometric analysis of the bands was performed through ImageJ Quant Software.

Fibroblast viability under starving conditions was assessed by MTT and a Trypan blue exclusion test following the manufacturer’s instructions.

### Immunofluorescent analysis of LC3B in primary skin fibroblasts

Fibroblasts were seeded at a density of 8 × 10^5^ cells in IF cover slips the day before starting experiments and incubated overnight at 37 °C, 5% CO_2_. The following day, autophagy was induced as indicated above and, at the end of the treatment, cells were washed in PBS and fixed in 4% paraformaldehyde for 10 min at R.T. Then, the cells were washed twice with PBS and incubated in PBS + 0,03% Triton + 3% BSA for 30 min at R.T. Thereafter, the cells were washed twice with PBS and incubated overnight at 4 °C with an anti- LC3B primary antibody. The following day, after two washings with PBS of 15 min at R.T. each, the cells were incubated with Alexa Fluor-conjugated specific secondary antibodies for 1 h at R.T. Finally, after two washings with PBS for 15 min at R.T. each, the coverslips were mounted and the images were taken with a Zeiss Axioplan 2 fluorescence microscope connected to a digital camera. The number of LC3B positive dots was quantified through a manual count of three independent observers.

Alternatively, healthy and RTT cells starved for 2 h in the absence of CQ were stained with a specific fluorescent dye which binds to the autophagosomal membrane (Cyto-ID Enzo Life Sciences). The overall procedure was performed according to the manufacturer’s instructions and, in this case, the cells were acquired at 40X magnification.

### Transmission Electron Microscopy

RTT patients enrolled in the study were analyzed by TEM. The figures presented are relative to the analyses performed on the patients carrying the R255X MeCP2 mutation. Additional patients were analyzed and are not included in the results. They carry the following MeCP2 mutations: T158M (2 patients), R270X (2 patients), P384 (early truncation), Exon-3 complete deletion and Exon-4 partial deletion, A168X, R294X, T327fs, plus one with unknown mutation. Blood samples were fixed with an equal volume of buffered Karnosky’s fixative. The samples were then dehydrated through an alcohol series and then infiltrated and embedded in a liquid EPON resin (Agar Scientific, Stansted Essex CM24 8GF United Kingdom), for morphological and ultrastructural analysis. After embedding, the resin blocks were then thin sectioned; sections of 50–70 nm thickness were collected on metal mesh ‘grids’ and stained with heavy metal solutions before observation in the TEM. All sections were examined by a Hitachi 7100 FA electron microscope.

### Citofluorimetric analyses

In order to stain RBCs, 5 µL of whole blood that was freshly isolated from RTT patients and healthy patients were diluted in saline buffer in the presence of an anti CD71 (1:200) and an anti COX-IV (1:200) antibody (Proteintech Group, Inc. U.S.A.) for 30 min at R.T. Thereafter, the cells were washed twice in PBS and then incubated with Alexa Fluor-conjugated specific secondary antibodies for 30 min at R.T.

An additional investigation by using Mitotracker Green (Molecular Probes) was performed by diluting the blood in saline buffer in the presence of 100 nM dye (30 min, room temperature). Data were acquired on a CytoFlex Cytometer and analysed with FlowJo software. Cell doublets were excluded using a pulse geometry gate (FSC-H × FSC-A).

### Immunofluorescent intracellular staining of RBC

Peripheral blood from healthy and RTT individuals (those bearing the MeCP2 R255X mutation) was harvested in tubes containing 2 mM EDTA and was centrifuged at 3500 rpm for 5 min. Then, the cells were washed four times with PBS supplemented with 2 mM EDTA and were centrifuged at 3000 rpm, 2 min, 4 °C. The cells were then re-suspended in PBS supplemented with 2 mM EDTA and 20% fetal bovine serum (ratio 5 μL cells: 200 μL buffer) and 200 μL of this suspension was spotted onto poly-L-lysine-coated slides (500 rpm for 10 min). The cells were fixed with 4% paraformaldehyde and incubated for 30 min at R.T. Thereafter, the cells were washed four times with PBS + 0,1% Tween (T-PBS) and permeabilized with PBS + 0,1% Triton for 10 min at R.T. Then, the cells were washed four times with T-PBS and incubated in a solution of 50% PBS and 50% High Contrast Diluent (Inova Diagnostics, USA) 30 min at R.T. to reduce the haemoglobin background. Finally, the cells were washed four times with T-PBS and incubated overnight at 4 °C with an anti COX-IV antibody (Proteintech Group, USA) and a CD71 antibody (Proteintech Group, USA). The following day, the unbound antibody was removed through four washes in T-PBS and the cells were incubated with Alexa Fluor-conjugated specific secondary antibodies for 1 h at R.T. After four washings with T-PBS to remove the unbound antibody, the coverslips were mounted. The images were acquired with a Zeiss Axioplan 2 fluorescence microscope connected to a digital camera.

### Western blotting analysis of RBCs

To search for the accumulation of mitochondrial proteins inside the RBCs of RTT patients, 4 × 10^6^ RBCs were withdrawn from the 3 RTT patients carrying the R255X MeCP2 mutation and from 2 healthy subjects.

The RBCs were lysed in the lysis buffer previously indicated for the fibroblast lysis procedure and kept on ice for 30 min followed by centrifugation at 13000 rpm for 30 min.

Thereafter, an equal volume of supernatant was loaded onto a 12% acryl-amide gel and analyzed by Western blotting using an anti-sirtuin3 antibody (Sigma-Aldrich, St. Louis, USA) and an anti-GAPDH antibody (Abcam, CO, UK).

### Experimental mice

#### Breeding

The *Mecp2* knockout mouse (*Mecp2* ^−/*y*^; strain B6.129 P(C) −*Mecp2* ^tm1.1Bird^/J Jax stock number: 003890) was used as a mouse model for RTT in this study. *Mecp2* mutant hemizygous males and heterozygous females were generated by crossing heterozygous females (*Mecp2* ^+/−^) with C57BL/6J wild type males, purchased from The Jackson Laboratory (Bar Harbor, ME, USA). Animals were kept in a temperature- und humidity-controlled 12 h light/dark cycle and had free access to water and standardized pellet food. Mice maintained under standard conditions and in accordance with Home Office regulations and licenses. Wild type littermates were used as controls. The animals were sacrificed and the tissues were recovered and stored at −80 °C. The national or institutional guidelines were used for the care and use of animals, and approval for the experiments was obtained from the ethical committees of the Italian Ministry of Health, and the UK Home Office.

#### Genotyping

Genomic DNA was extracted from ear clips or tail tips of pups. The genotype of the mice was determined by polymerase chain reaction using PCR primers and following the conditions described in the web site of the Jackson Laboratories (USA).

#### Scoring of symptoms

Mice were scored on a weekly basis for a number of symptoms arising from Mecp2 deficiency as reported^49^.

#### Brain collection

After transcardial perfusion with saline, brains were removed and bisected on the sagittal plane. Brain hemispheres were immediately frozen in dry ice and stored at −80 °C until assay. At the time of the assays, brain was homogenized (10% W/V) in phosphate-buffered saline (PBS), pH 7.4.

### Statistical analysis

A one-way analysis of variance (ANOVA) was used to assess statistically significant differences among groups and Tukey’s honest significance post hoc test was used for pairwise comparisons after the analysis of variance.

An unpaired student test was also used when indicated.

### Ethic Committee Approval

All methods were carried out in accordance with relevant guidelines and regulations. Furthermore, the enrolment of the patients as well as the experimental protocols adopted in the study were approved by the local ethic committee (University of Rome Tor Vergata) (protocol: 0028735/2015). An informed consent was obtained from all subjects.

## Electronic supplementary material

Supplementary Information
