# Author Correction: Retention of Mitochondria in Mature Human Red Blood Cells as the Result of Autophagy Impairment in Rett Syndrome

**DOI:** 10.1038/s41598-021-91550-3

**Published:** 2021-06-07

**Authors:** Diego Sbardella, Grazia Raffaella Tundo, Luisa Campagnolo, Giuseppe Valacchi, Augusto Orlandi, Paolo Curatolo, Giovanna Borsellino, Maurizio D’Esposito, Chiara Ciaccio, Silvia Di Cesare, Donato Di Pierro, Cinzia Galasso, Marta Elena Santarone, Joussef Hayek, Massimiliano Coletta, Stefano Marini

**Affiliations:** 1 Department of Clinical Sciences and Translational Medicine, University of Rome Tor Vergata, Rome, Italy; 2 Department of Biomedicine and Prevention, University of Rome Tor Vergata, Rome, Italy; 3 Department of Life Sciences and Biotechnology, University of Ferrara, Ferrara, Italy; 4 Plant for Human Health Institute, North Carolina State University, Kannapolis, NC USA; 5 Department of Medicine of Systems, University of Tor Vergata, Rome, Italy; 6 Neuroimmunology Unit, Santa Lucia Foundation, Rome, Italy; 7 Institute of Genetics and Biophysics “A.Buzzati Traverso”, Naples, Italy; 8 IRCCS Neuromed, Pozzuoli, (Is) Italy; 9 University Department of Pediatrics, Bambino Gesù Children’s Hospital, University of Rome Tor Vergata, Rome, Italy; 10 Child Neuropsychiatry Unit, University Hospital, Azienda Ospedaliera Universitaria Senese (AOUS), Siena, Italy

Correction to: *Scientific Reports* 10.1038/s41598-017-12069-0, published online 26 September 2017.

In the original version of this Article, the author Silvia Di Cesare was incorrectly indexed.

The original Article has been corrected.

