# Supplementary material for: Retention of Mitochondria in Mature Human Red Blood Cells as the Result of Autophagy Impairment in Rett Syndrome
Source: Sci Rep. 2017 Sep 26;7:12297. doi: 10.1038/s41598-017-12069-0 (PMC5614985; doi:10.1038/s41598-017-12069-0)
Supplement: Supplementary file 1 — Supplementary Information [file 41598_2017_12069_MOESM1_ESM.doc]

**Supplementary Figures**

**Retention of Mitochondria in Mature Human Red Blood Cells as the Result of Autophagy Impairment in Rett Syndrome**

Diego Sbardella, Grazia Raffaella Tundo, Luisa Campagnolo, Giuseppe Valacchi, Augusto Orlandi, Paolo Curatolo, Giovanna Borsellino, Maurizio D’Esposito, Chiara Ciaccio, Silvia Di Cesare, Donato Di Pierro, Cinzia Galasso, Marta Elena Santarone, Joussef Hayek, Massimiliano Coletta, Stefano Marini.


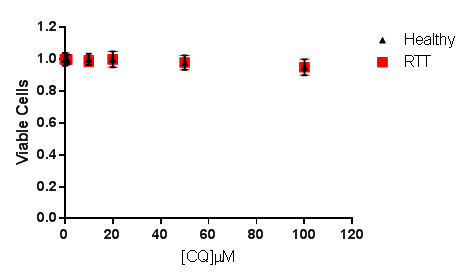


**Figure 1**: **CQ administration has no effect on fibroblasts viability**

Healthy and RTT fibroblasts were allowed to grow in standard medium in the presence of different CQ concentrations ranging from 10 μM to 100 μM for 6h. Viability of the cells was measured by MTT assay. The data reported are expressed as the percentage of live cells *vs* the number of cells at time 0, i.e. before the administration of CQ. Results presented are the means ± S.E. of five independent experiments performed in triplicate. No significant differences were observed between the groups at the various time-points (oneway ANOVA, followed by Tukey’s test, n =15).


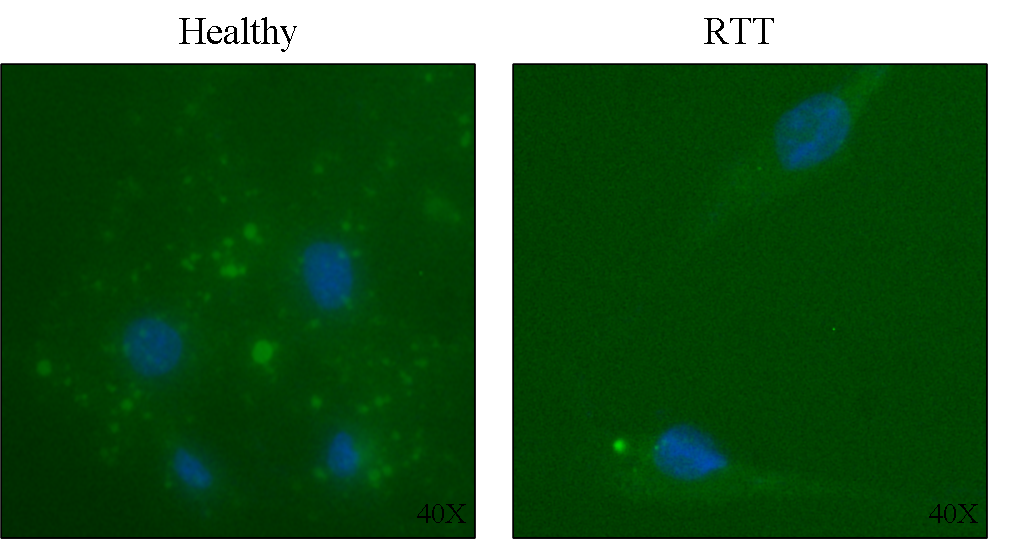


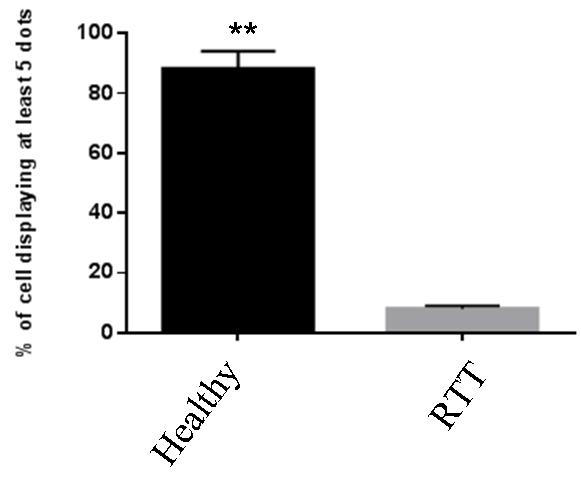


**Figure 2**: **impaired autophagosomes formation in RTT fibroblasts**

Healthy and RTT fibroblasts (n = 2 for both cases) starved for 2 h in the absence of CQ were stained with the Cyto-ID dye which specifically targets the autophagosomes (*higher panel*). Images were acquired through an immuno-fluorescence microscope at 40X.

*Lower panel*: percentage of cells displaying at least 5 Cyto-ID positive dots. The results are the means ± S.E. of three independent experiments. **, significantly different from control (** *p* <0.001, unpaired τ Student’s test, *n* = 6).


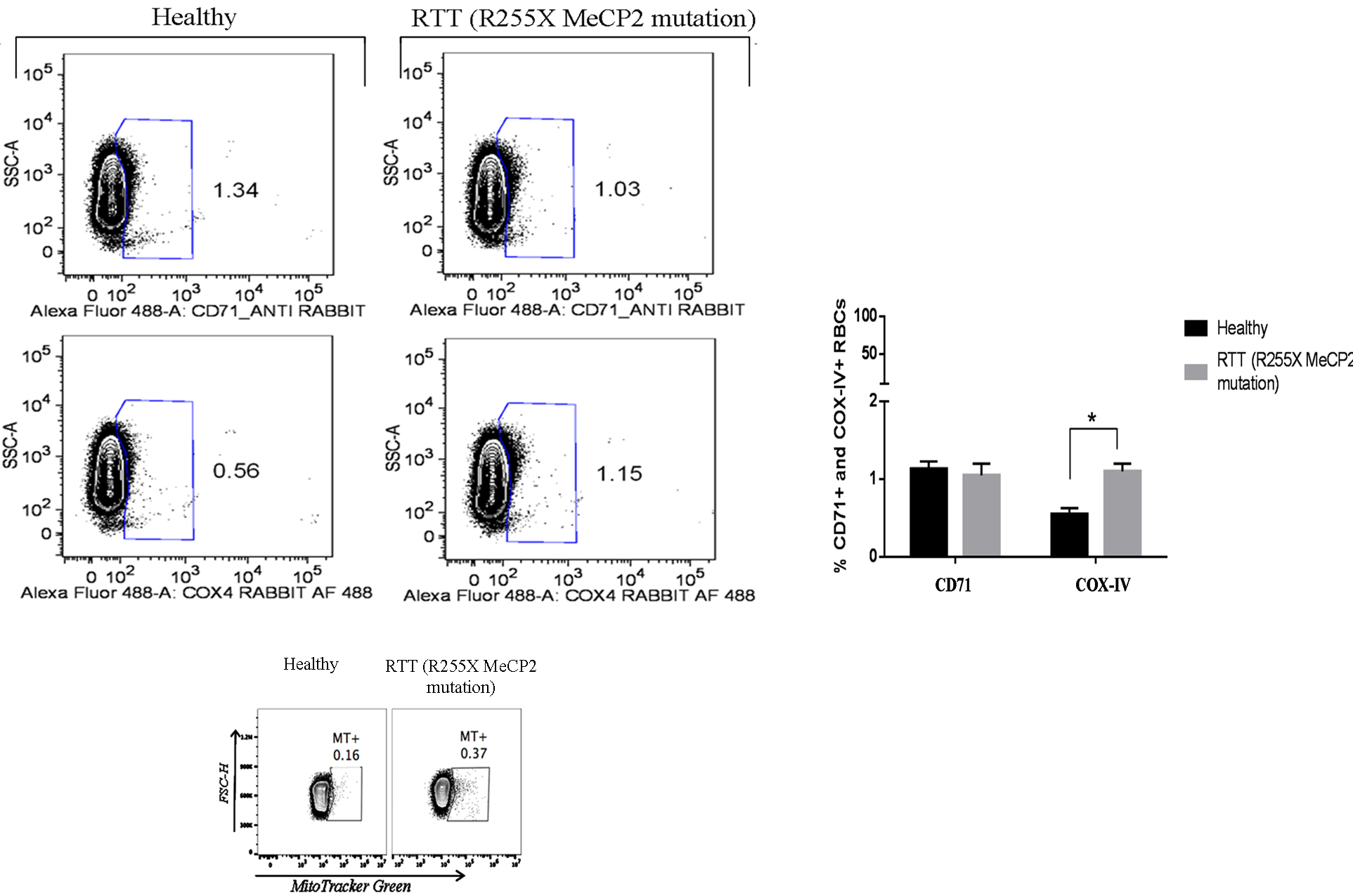


**Figure 3: staining of RBCs retaining mitochondria by cytofluorimetry**

*upper panel*: blood cells from healthy and RTT (bearing the R255X MeCP2 mutation) patients were indirectly stained with anti-human CD71 and anti-human COX-IV antibodies according to standard protocols. Cell populations were considered as positive compared to the autofluorescence observed in presence of the secondary antibody alone. The statistical analysis of the mean of three independent observations on the same blood specimen from the healthy (n = 3) and RTT (bearing the R255X MeCP2 mutation, n = 3) regarding the percentage of CD71^+^ and COX-IV^+^ RBCs indicated that the difference in the CD71^+^ RBCs were not significant between the two groups, whereas the difference in the COX-IV^+^ RBCs was significant for * p<0.01 (unpaired τ Student’s test). Blood cells from healthy (n = 1) and RTT (bearing the R255X MeCP2 mutation, n = 1) patients were further stained with Mitotracker green (100 nM). One single case is shown (*lower panel*).


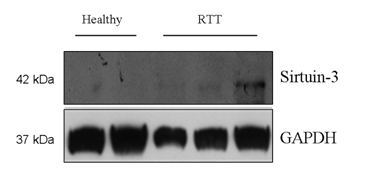

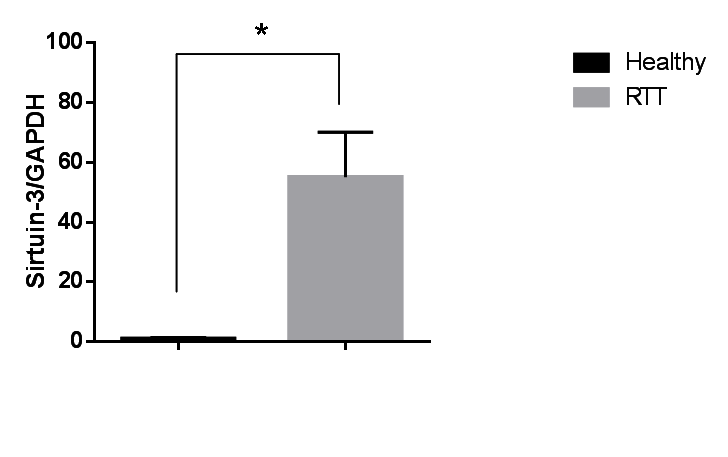


**Figure 4**: **detection of sirtuin-3 in lysates from RTT RBCs**

lysates from RBCs isolated from healthy subjects (n = 2) and RBCs isolated from the RTT patients carrying the R255X MeCP2 mutation (n = 3) were analyzed by Western blotting. An anti-sirtuin3 antibody recognized a 42 kDa protein corresponding to the mitochondrial marker exclusively in the RTT lysates. GAPDH was used as internal control. The sirtuin-3/GAPDH ratio was significantly increased for the three RTT patients with respect to the healthy patients. Results presented are the means ± S.E. of three independent experiments (* significantly different from control, p<0.001, unpaired τ Student’s test).
